# Ensemble Dictionary Learning for Single Image Deblurring via Low-Rank Regularization

**DOI:** 10.3390/s19051143

**Published:** 2019-03-06

**Authors:** Jinyang Li, Zhijing Liu

**Affiliations:** School of Computer Science and Technology, Xidian University, Xi’an 710071, China; liuzhijing@vip.163.com

**Keywords:** image deblurring, low-rank constraint, self-repetitive structures, nonlocal similarity, ensemble dictionary learning

## Abstract

Sparse representation is a powerful statistical technique that has been widely utilized in image restoration applications. In this paper, an improved sparse representation model regularized by a low-rank constraint is proposed for single image deblurring. The key motivation for the proposed model lies in the observation that natural images are full of self-repetitive structures and they can be represented by similar patterns. However, as input images contain noise, blur, and other visual artifacts, extracting nonlocal similarities only with patch clustering algorithms is insufficient. In this paper, we first propose an ensemble dictionary learning method to represent different similar patterns. Then, low-rank embedded regularization is directly imposed on inputs to regularize the desired solution space which favors natural and sharp structures. The proposed method can be optimized by alternatively solving nuclear norm minimization and $l_{1}$ norm minimization problems to achieve higher restoration quality. Experimental comparisons validate the superior results of the proposed method compared with other deblurring algorithms in terms of visual quality and quantitative metrics.

## 1. Introduction

Image blurs are the most common artifacts that appear in consumer-level photographing and other imaging sensors [1,2,3,4]. They are usually caused by relative motion between the camera and the scene, e.g., camera shake and its removal. The results they caused will lead to significant image degradation that affect the performance of computer vision such as image super-resolution, image quality assessment, collaborative tracking, object recognition and detection, etc. Therefore, image deblurring, one of the most fundamental problems in computer vision, has been studied extensively and advanced rapidly in the past decades.

The theory of sparsity can be dated back to the problems of variable analysis and selection which discussed in [5] in 1970s. Since then, sparse representation has been widely exploited and is now known to be a powerful image restoration technique [6,7]. The classic sparse representation is mathematically modeled so that signals (image patches in this paper) can be reconstructed by linearly combining a number of dictionary atoms: y=Dα+n, where $y\in R^{n}$ denotes an image patch to be represented, *D* ∈ $R^{n\times L}$ and n≪L is an over-complete dictionary which consists of *L* prototype signal-atoms, α represents the coefficient vector with dimension *L*, and *n* is observation noise. Under the framework of sparse representation, statistical model of sparse coefficients and dictionary learning are the two most related issues.

The study of the former rapidly sparkled the interest in sparse coding. Image restoration aims at restoring a high-quality image from its degraded (e.g., low-resolution, noisy and blurred) measurements. Considering the ill-posed property of image restoration, prior knowledge of natural image is required to regularize the desired solution under the observation model. Exploiting and modeling appropriate prior is critical to the success of image restoration, and hence various of regularizers have been developed to model realistic situation for real world signals. As an alternative, statistical models of sparse coefficients (i.e., sparsity prior) have been extensively employed based on the discovery that many types of signals (e.g., image patches) can be represented using a small number of structural primitives that sparsely chosen out of a basis function [8,9] (e.g., over-complete dictionary).

Standard image restoration algorithms recover the unknown image patch by imposing the sparsest coefficient vector α that satisfies $\alpha \;=\;\min_{\alpha }{∥\alpha ∥}_{0},s.t.{∥y-D\alpha ∥}_{2}\leq \varepsilon$, where ${∥•∥}_{0}$ is $l_{0}$ norm counting the number of nonzero elements of α, ε is a small number. The reconstructed image (e.g., deblurred image) of degraded observation *y*, denoted by $\hat{x}$, then can be estimated by $\hat{x}\approx D\alpha$. However, optimizing with $l_{0}$ minimization is difficult because it is both NP-hard and unstable in the presence of observation noise. To address this, Donoho et al. [10] has proved that the non-convex $l_{0}$ norm can be replaced by its convex $l_{1}$ counterpart under certain conditions—namely: $\alpha \;=\;\min_{\alpha }{∥\alpha ∥}_{1},s.t.{∥y-D\alpha ∥}_{2}\leq \varepsilon$, where ${∥•∥}_{1}$ is $l_{1}$ norm counting the sum of the absolute values of each element in α. The $l_{1}$ norm minimization is widely used to impose sparse regularization and can be efficiently solved by an iterative shrinkage algorithm [11], augmented Lagrange multiplier method [12], and Bregman iteration algorithm [13]. Elhamifar and Vidal et al. [14] presented a subspace clustering algorithm for segmenting multiple motions in video. In this algorithm, they use $l_{1}$ optimization to obtain sparse representation and then apply the sparse representation to spectral clustering to obtain the segmentation. Recently, a novel approach has theoretically and experimentally shown that more exact reconstruction results can be achieved with fewer dictionary atoms by replacing the $l_{1}$ norm with $l_{p}$ norm with 0≤p≤1 [15]. However, since the $l_{p}$ norm is non-convex, the optimizing task of $l_{p}$ norm minimization is time consuming and computationally complex.

The nonlocal self-similarity constraint is one of the most commonly used regularization for image restoration [13,16]. The key motivation lies in the observation that natural images are full of self-repetitive structures. By estimating more accurate sparse coefficients, sparse coding noise can be suppressed and image restoration performance can be improved. In [17], a two-step image deblurring algorithm based on nonlocal model has been presented by collaborating with hard-thresholding and a regularized Wiener version of BM3D. Dong et al. [6] developed a nonlocal Gaussian scale mixture (GSM) model for image restoration. In this method, the sparse coefficients and their variances can be iteratively calculated by the method of alternating minimization. In [18], a just-noticeable defocus (JNB) algorithm is presented for tiny defocus blur analysis. However, the performance of the JNB method will severely decrease due to inaccurate estimation of sparse coefficients. To make this procedure more reliable and stable, Li et al. [19] proposed to learn the non-zero mean I.I.D. Laplacian distribution for sparse coefficients by utilizing nonlocal similarity. Greatly improved performance for defocus blur estimation is achieved in [19].

A low-rank constraint exploits the spatial redundancy of natural images and estimates parameters of natural image patches from both local and nonlocal information [7,20]. Low-rank regularization can be approximated and formulated as a nuclear norm which equals the sum of the singular values of the objective matrix. Liu et al. [21] presented a low-rank representation method, which seeks the lowest rank representation among all the candidates, to cluster the samples into the respective subspaces. Since the optimizing of nuclear norm minimization can be efficiently solved by singular value decomposition (SVD), sparse representation via low-rank regularization has been successfully applied to various image restoration applications.

The dictionary learning includes online dictionary learning, over-complete dictionary learning, multiscale dictionary learning and adaptive dictionary learning. Mairal et al. [22] proposed to construct an online dictionary learning by utilizing stochastic approximations. Elad and Aharon et al. [7,23] proposed the K-SVD methods which learned an over-complete dictionary. In [18], a JNB algorithm is presented for tiny defocus blur analysis using a pre-trained over-complete dictionary. However, the over-complete dictionary is rank-deficient, which leads to a constrained solution space and high computational complexity. A multiscale dictionary learning algorithm has been presented in [24] for sparse representation by utilizing an efficient quadtree decomposition. However, this method follows the K-SVD method by using over-complete dictionary, which implies that it suffers from the same limitation. Ravishankar and Bresler [25] presented an adaptive dictionaries learning framework from k-space data for compressed sensing magnetic imaging (CSMRI). An algorithm of adaptive sparse domain selection and adaptive regularization [26] for image deblurring has been proposed. Compared with other sparse representation models that are based on over-complete dictionary, the dictionaries trained in [26] over patches which were gathered using nonlocal similarity, can increase the accuracy of patch representation and decrease computational complexity. Since each subdictionary $D_{i}$ in the dictionary set is trained over the patches from the *i*-th cluster, subdictionary $D_{i}$ can represent the pattern similar with the *i*th cluster. Besides, it also implies that the subdictionary can propagate structural information to input patches (with similar structure). Under this assumption, the similar patches can be formulated as $X_{i}\approx D_{i}A_{i}$ to connect visual similar features and their basis representations, where $X_{i}$ denotes a patch set containing similar patches with the *i*-th cluster, $A_{i}$ is coefficient matrix for $X_{i}$. However, gathering similar patches from degraded input patch set, denoted by $Y_{i}$, only by patch clustering is less effective—especially given the fact that the input is suffering from noise, blur, or other visual artefacts. Hence, a fundamental problem remains open: how to regularize the degraded input for sparse coefficients over a specific dictionary learned from sharp and clear image datasets? This problem is usually solved by imposing high-pass filtering or other image restoration algorithms. But the performance becomes susceptible when it is tangled with other programs.

In this paper, we propose an ensemble dictionary learning method via low-rank constraint for image deblurring. Considering the fact that it is difficult and unstable to represent blurred features over sharp primitives, the structures of training data hence can not be directly reused for image deblurring task. For this reason, we propose to utilize low-rank embedded regularization, which is directly imposed on inputs, for properly linking structural features with sharp primitives. We then propose to learn an ensemble dictionary set to represent different similar patterns under the observation that blurred images consist of rich repetitive structures. Different from previous approaches, the proposed method can much improve the performance and stability of image deblurring by directly regularizing inputs rather than imposing other image restoration algorithms or filters. First, we learn an ensemble dictionary set as prototypes and design a coarse-grained patch clustering for the characterization of nonlocal similarity. Second, we impose fine-grained low-rank regularization for gathering more informative structurally similar features. Lastly, solutions can be obtained by alternatively optimizing two sub-problems.

The paper is organized as follows. Section 2 describes the details of patch clustering and ensemble dictionary learning, sparse representation model via low-rank constraint and optimization, respectively. In Section 3, qualitative and quantitative experimental comparisons with other algorithms are presented.

## 2. The Proposed Method

**Figure 1:**
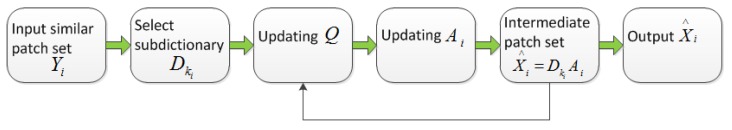
The pipeline of our proposed framework.

In this paper, we present a low-rank constrained, ensemble dictionary learning model for single image deblurring. Under the framework of sparse representation, the proposed method consists of three components: coarse-grained patch clustering for nonlocal similarity characterization and ensemble dictionary learning; fine-grained low-rank regularization for linking structural features with sharp primitives; and a sparsity constraint for sparse coefficients. Figure 1 illustrates the whole pipeline of the proposed framework, where $\hat{X_{i}}$ denotes the reconstructed patch set corresponding to $Y_{i}$.

### 2.1. Patch Clustering and Ensemble Dictionary Learning

The assumption that natural images consist of rich self-repetitive structures has been widely adopted in various image restoration tasks and applications. In this subsection, we first utilized a coarse-grained patch clustering algorithm to obtain nonlocal similarity, then introduce an effective ensemble dictionary learning method for sparse coefficients. For each blurred image patch, denoted by $y_{i}$, the proposed method constructs a patch set—denoted by $Y_{i}\;=\;\left[y_{i,1},y_{i,2},\;\cdots ,y_{i,m}\right]\in R^{n\times m}$—containing *m* patches similar to $y_{i}$ (including $y_{i}$ itself). Typically, the patch set was obtained by utilizing a clustering algorithm (e.g., the KNN method) in a larger window centered at *i*.

Recent years have witnessed the popularity and effectiveness of dictionary learning-based sparse coding approaches for image deblurring. Most state-of-the-art methods use either an over-complete dictionary, or an updated dictionary. However, the performance of these methods is unstable and easily influenced by the following issues. First, the over-complete dictionary can only represent a limited class of structures [26]—this is insufficient, as structures and contents usually vary significantly across different images or patches within them. Secondly, solving and optimizing the minimization task over an over-complete dictionary is mathematically difficult, since the over-complete dictionary is rank-deficient, the solution cannot be converged. Last, dictionary atom updated during the iteration process can easily fall into local minima that will decrease performance in subsequent stages and negatively impact the final results.

To address these problems, a discriminative dictionary (or dictionary set) was needed to represent various classes of image structures and simultaneously decrease computational complexity. Similar to [19], we adopted principal component analysis (PCA) [27] to construct a set of ensemble dictionary, denoted by $D_{k}\left(k\;=\;1,\cdots ,K\right)$, and the corresponding centroid, denoted by $\mu_{k}$. Different from [19], we selected 1000 natural, yet sharp images from authoritative datasets (e.g., ImageNet). We cropped from them and 100,000 image patches are obtained. Subsequently, the proposed method created a training set of the image patches with an intensity variance greater than a predefined threshold. The purpose was to obtain sharp and meaningful structures and exclude blurred and smooth patches. For each patch, $y_{i}$, and patch set, $Y_{i}$, the dictionary from the dictionary set can be selected by

(1) $$k_{i}=\underset{k}{\arg\min}\;{∥y_{i}-\mu_{k}∥}_{2}.$$

However, it is unstable and inefficient to directly compare the distance between $y_{i}$ and $\mu_{k}$, since the original input $y_{i}$ suffers from blur and noise, the distance between $y_{i}$ and $\mu_{k}$ could be great as $\mu_{k}$ is the centroid obtained from a training set within which the training samples are sharp and clear image patches. To solve this problem, a projection matrix is employed to map the distance between $y_{i}$ and $\mu_{k}$ into a new subspace; thus the accuracy of the selected index (i.e., $k_{i}$) can be greatly improved. Let $M\;=\;\left[\mu_{1},\mu_{2},\;\cdots ,\mu_{K}\right]\in R^{n\times K}$ be a matrix whose each column denotes a centroid. Then, an orthogonal PCA transformation matrix, denoted by $\tilde{\Omega }$, can be achieved by applying PCA to the co-variance matrix of *M*. Similar to the learning process of ensemble PCA dictionaries, only the eigenvectors that correspond to the first several largest eigenvalues are used to construct the compact and effective projection matrix, Ω. The dictionary selection can be modeled as

(2) $$k_{i}=\underset{k}{\arg\min}\;{∥\Omega \left(\hat{y_{i}}-\mu_{k}\right)∥}_{2},$$

where $\hat{y_{i}}$ is the high-pass filtered vision of $y_{i}$.

### 2.2. Sparse Representation Model via Low-Rank Constraint

Low-rank prior is known as a data-authentic prior and widely employed in sparsity constrained image restoration tasks. A digital image is made up of pixels with certain length and width. Mathematically, each image can be formed as an image matrix and within which each pixel can be regarded as an element of the matrix. Image patches, represented as vectors, can be obtained by cropping from the image. As illustrated in Section 2.1, natural images consist of rich self-repetitive structures suggesting that the image matrix rank should be relatively low. However, due to the influence of noise or blur, the information obtained by patch clustering algorithms is insufficient. As such, we employ low-rank regularization, denoted by *Q*, directly on input patch set to properly link structural features with the pre-trained ensemble dictionary set. By doing so, similar features and patterns in input patch set can be mapped to the coefficient matrix over the specific dictionary

(3) $$\underset{Q,A_{i}}{\arg\min}∥QY_{i}-D_{k_{i}}A_{i}∥{}_{F}^{2}+\lambda rank\left(Q\right)+\gamma \Phi \left(A_{i}\right)$$

where $Q\in R^{n\times n}$ is low-rank projection which is mathematically denoted as a matrix, $Y_{i}$ denotes the image patch set constructed via a given patch $y_{i}$, $\alpha_{i,j}$ is a sparse coefficient for $y_{i,j}\left(j=1,\cdots ,m\right)$ and denoted by $A_{i}\;=\;\left[\alpha_{i,1},\alpha_{i,2},\;\cdots ,\alpha_{i,m}\right]$ the coefficient matrix for $Y_{i}$, $D_{k_{i}}$ represents the compact dictionary with the index $k_{i}$ obtained by Equation (2) for image patch $y_{i}$ and patch set $Y_{i}$, rank(•) denotes rank regularization for a matrix, Φ(•) is the sparsity operator of a matrix, λ and γ are trade-off parameters for the two regularization terms.

Inspired by the study [20], we exploited the low-rank constraint to build a mapping matrix that was able to obtain fine-grained informative structures. Generally, the rank(Q) will be employed by nuclear norm ${∥Q∥}_{*}$—i.e., the sum of the singular values of matrix *Q*—as a convex surrogate to solve rank minimization. The proposed method for single image deblurring can be formulated as

(4) $$\underset{Q,A_{i}}{\arg\min}∥QY_{i}-D_{k_{i}}A_{i}∥{}_{F}^{2}+\lambda {∥Q∥}_{*}+\gamma \sum_{j\;=\;1}^{m}{∥\alpha_{i,j}∥}_{1},$$

where $\sum_{j\;=\;1}^{m}{∥\alpha_{i,j}∥}_{1}$ represent the sparsity prior regularization term.

### 2.3. Optimization for the Proposed Regularization

The proposed model for Equation (4) can be optimized and solved by alternatively solving

(5) $$\underset{Q}{\arg\min}∥QY_{i}-D_{k_{i}}A_{i}∥{}_{F}^{2}+\lambda {∥Q∥}_{*},$$

and

(6) $$\underset{\alpha_{i,j}}{\arg\min}∥QY_{i}-D_{k_{i}}A_{i}∥{}_{F}^{2}+\gamma \sum_{j\;=\;1}^{m}{∥\alpha_{i,j}∥}_{1}.$$

We are able to solve Equations (5) and (6) by alternatively minimizing *Q* and $A_{i}$ with the other variable fixed.

#### 2.3.1. Updating *Q* by Fixing $A_{i}$

In this step, we fix $A_{i}$ and update *Q*. With $A_{i}$ fixed, Equation (5) is a low-rank minimization problem and can be solved by an augmented Lagrange multiplier method [12,28]. In order to optimize Equation (5) effectively, an auxiliary variable, *V*, is introduced to guide the solution. The objective function in Equation (5) can be converted to the following equivalent formulation

(7) $$\min_{Q,V}∥QY_{i}-D_{k_{i}}A_{i}∥{}_{F}^{2}+\lambda {∥V∥}_{*}\;s.t.\;Q\;=\;V.$$

The augmented Lagrangian function then can be derived from

(8) $$∥QY_{i}-D_{k_{i}}A_{i}∥{}_{F}^{2}+\lambda {∥V∥}_{*}+\langle R,Q-V\rangle +\frac{\phi }{2}∥Q-V∥{}_{F}^{2},$$

where $\langle •\rangle$ denotes inner product operator of the matrix, *R* represents the Lagrange multiplier, and ϕ>0 denotes the regularization parameter.

The updated *Q* and *V* at iteration p+1 can be estimated as follows

(9) $$V_{p+1}\;=\;\underset{V}{\arg\min}\frac{1}{2}∥V-Q_{p}-\frac{R_{p}}{\phi_{p}}∥{}_{F}^{2}+\frac{\lambda }{\phi_{p}}{∥V∥}_{*}$$

which can be solved by a singular value thresholding algorithm [29].

(10) $$\begin{array}{cc} Q_{p+1} & =\underset{Q}{\arg\min}∥QY_{i}-D_{k_{i}}A_{i}∥{}_{F}^{2}+\langle R_{p},Q-V_{p+1}\rangle +\frac{\phi_{p}}{2}∥Q-V_{p+1}∥{}_{F}^{2}\\ & \;=\;\left(2D_{k_{i}}A_{i}Y_{i}^{⊤}+\phi_{p}V_{p+1}-R_{p}\right){\left(2Y_{i}Y_{i}^{⊤}+\phi_{p}I_{n}\right)}^{-1}, \end{array}$$

where $I_{n}\in R^{n\times n}$ denotes the identity matrix.

#### 2.3.2. Updating $A_{i}$ by Fixing *Q*

With the given *Q*, Equation (6) is a conventional $l_{1}$ minimization problem which can be efficiently solved by an iterative shrinkage thresholding algorithm [30,31,32].

At iteration t+1

(11) $$\begin{array}{c} \alpha_{i,j}^{\left(t+1\right)}\;=\;soft\left(\alpha_{i,j}^{\left(t\right)},\iota_{i,j}\right), \end{array}$$

where $soft(•,\iota_{i,j})$ is a soft thresholding function whose threshold is $\iota_{i,j}$.

## 3. Experimental Results and Evaluations

### 3.1. Comparison with State-of-the-Art Methods

In this section, we briefly describe the methods used for comparison. Xu’s method [33] generalizes a new loss function to $l_{0}$ sparse representation for approximating $l_{0}$ sparsity and decreasing energy. In Shen’s method [34], the blur map is first generated by local contrast prior and guided filter. Subsequently, the spatially varying deblurring algorithm is solved by L1−2 optimization. Last, scale selection is adopted to remove ringing artifacts from the output. Yang’s method [35] handles multichannel deblurring by minimizing the sum of total variation (TV) of a multichannel and a data fidelity term. An adaptive sparse domain selection and adaptive regularization algorithm [26] for image deblurring has been proposed. In [26], each compact subdictionary is trained by patches gathered with nonlocal similarity. Dong et al. [36] proposed a centralized sparse representation (CSR) model for image deblurring. In this model, local and nonlocal sparsity constraints are unified for sparse coding.

### 3.2. Comparisons and Evaluations

In this section, the proposed method is experimentally evaluated using a blurry image dataset [37] and 36 blurred images including defocus blur and motion blur (i.e., camera rotation and translation) that captured by hand-held cameras (e.g., mobile phone and SLR camera). We compared the proposed method with other recent approaches by directly running their public executable programs or source codes. All comparisons are implemented in MATLAB on a PC with an Intel Xeon E5-2670 CPU and 64GB RAM. Similar to [19], we formed a 64-dimension vector by extracting each image patch with size 8×8, and trained the ensemble dictionary set on over 100,000 meaningful image patches—cropped from 1000 sharp, yet natural images based on a selection criterion. In all experiments, the parameters were set as follows: n=64, m=20, K=220, λ=0.18 and γ=0.68. The choice of *n*, *m* and *K* was similar to our previous work [19]. λ and γ are empirically setted.

**Figure 2:**
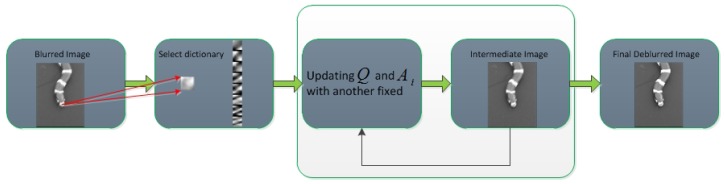
Main step of the proposed framework.

Figure 2 summarizes the main step of the proposed method which is illustrated with a simple input. For each blurred image patch in a blurred image (e.g., patch pointed by two arrows), the index of the dictionary from the pre-learned ensemble dictionary set can be obtained by Equation (2), for simplicity, we only show one atom of the selected dictionary. Then, followed by the procedures described in Section 2, the output can be iteratively optimized until convergence.

**Figure 3:**
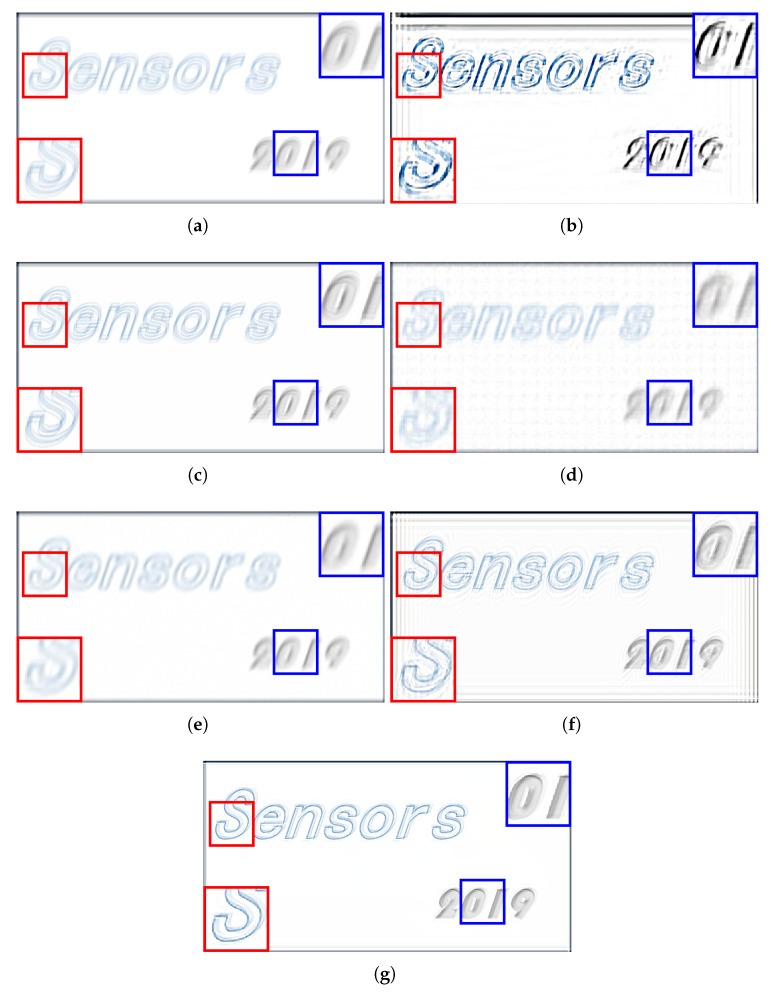
Comparison of different deblurring algorithms using a motion blurred image. (**a**) the input blur image; (**b**) the deblurring result of Xu’s method [33] (peak signal-to-noise ratio (PSNR) = 14.76, structural similarity index (SSIM) = 0.72); (**c**) the deblurring result of Shen’s method [34] (PSNR = 20.06, SSIM = 0.88); (**d**) the deblurring result of Yang’s method [35] (PSNR = 22.26, SSIM = 0.84); (**e**) the deblurring result of Dong’s method [26] (PSNR = 22.01, SSIM = 0.87); (**f**) the deblurring result of Wiener filter (PSNR = 22.98, SSIM = 0.80); (**g**) the deblurring result of proposed method (PSNR = 25.00, SSIM = 0.91).

**Figure 4:**
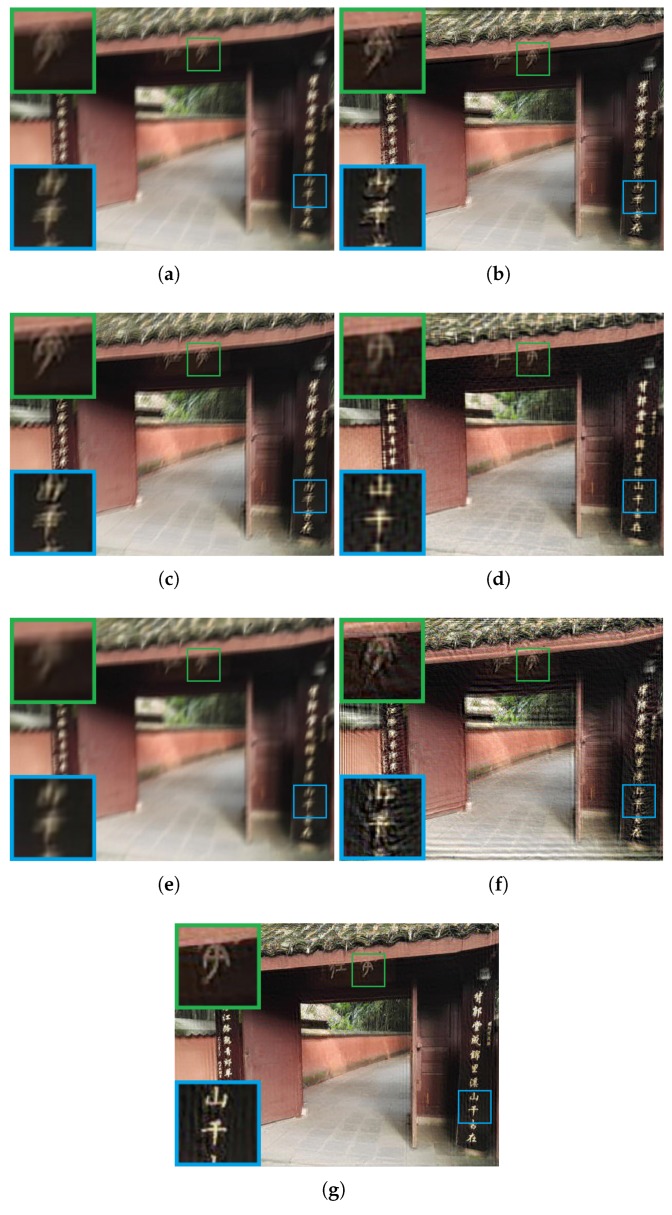
Comparison of different deblurring algorithms using a motion blur and continuous defocus blur image. (**a**) the input blur image; (**b**) the deblurring result of Xu’s method [33] (PSNR = 17.34, SSIM = 0.58); (**c**) the deblurring result of Shen’s method [34] (PSNR = 33.13, SSIM = 0.94); (**d**) the deblurring result of Yang’s method [35] (PSNR = 23.28, SSIM = 0.68); (**e**) the deblurring result of Dong’s method [26] (PSNR = 36.34, SSIM = 0.95); (**f**) the deblurring result of Wiener filter (PSNR = 16.56, SSIM = 0.41); (**g**) the deblurring result of proposed method (PSNR = 38.70, SSIM = 0.96).

**Figure 5:**
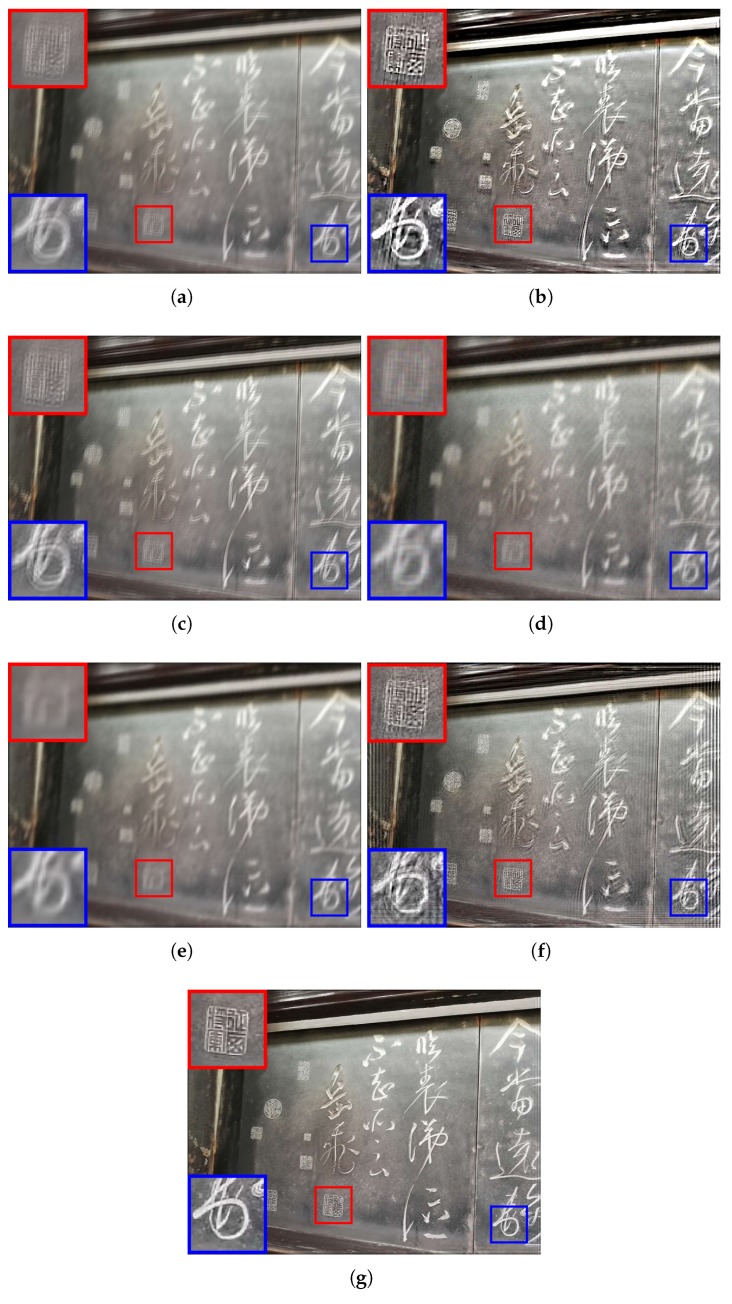
Comparison of different deblurring algorithms using a motion blur and continuous defocus blur image. (**a**) the input blur image; (**b**) the deblurring result of Xu’s method [33] (PSNR = 17.55, SSIM = 0.43); (**c**) the deblurring result of Shen’s method [34] (PSNR = 22.04, SSIM = 0.57); (**d**) the deblurring result of Yang’s method [35] (PSNR = 21.72, SSIM = 0.54); (**e**) the deblurring result of Dong’s method [26] (PSNR = 21.91, SSIM = 0.57); (**f**) the deblurring result of Wiener filter (PSNR = 21.16, SSIM = 0.51); (**g**) the deblurring result of proposed method (PSNR = 23.40, SSIM = 0.62).

**Figure 6:**
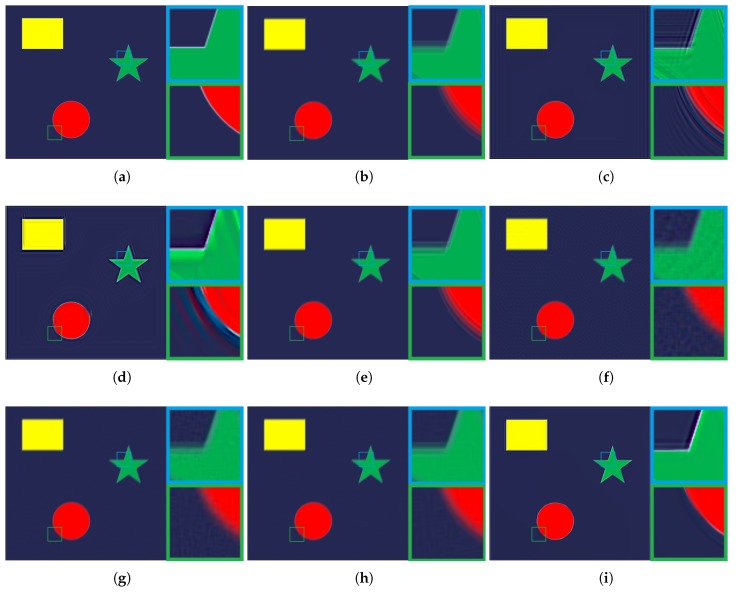
Comparison of different deblurring algorithms using a synthesis blurry image. (**a**) the original sharp image; (**b**) the blurry image obtained by a convolution between (**a**) and a blur kernel; (**c**) the deblurring result of Wiener filter; (**d**) the deblurring result of Xu’s method [33]; (**e**) the deblurring result of Shen’s method [34]; (**f**) the deblurring result of Yang’s method [35]; (**g**) the deblurring result of Dong’s method [26]; (**h**) the deblurring result of CSR method [36]; (**i**) the deblurring result of proposed method.

The experimental comparisons were qualitatively and quantitatively evaluated on visual quality, peak signal-to-noise ratio (PSNR), and structural similarity index (SSIM). PSNR is the ratio between the maximum power of the restored image and the power of its blurred input. While SSIM is used for measuring the similarity between the input blurred image and its restored version. To evaluate the performance of visual comparisons, qualitative results of each method are shown in Figure 3, Figure 4, Figure 5 and Figure 6.

Figure 3 shows the compared test results on a motion blur image. We can see that the result of Xu’s method [33] can hardly remove the blur effect, besides, ringing artifacts (i.e., black lines along the image) and pixels drift phenomena can be detected in the entire image. In Figure 3c, the performance is barely satisfactory since the blur is less removed. Figure 3d,e show that Yang’s method and Dong’s method can remove some blur effect. However, orderly arranged pixel clusters, which can be assumed as noises, can be found in the entire image in Yang’s result. Although Dong’s method can remove some blur effect, the decreased contrast still makes the visual effect uncomfortable. Besides, the texture in both Yang’s result and Dong’s result is still hard to identify. The deblurring result of Wiener filter described in Figure 3f can better restore some structures of the content. Compared with other competing algorithms, Wiener filter can obtain a better visual quality by achieving a higher contrast ratio. However, the result of Wiener filter, as well as the result of Xu’s method [33], contains a large number of ringing artefacts which decrease the performance of deblurring. As a contrast, the visual performance of the proposed method is better than all compared method.

Figure 4 and Figure 5 show two examples of images that suffer from motion blur and continuous defocus blur. Both images were captured by a HUAWEI cell phone. As shown in Figure 4b, there exists ringing artifacts and pixel drift phenomena that lead to a reduction in visual quality. In Figure 4c, although the visual quality is improved in comparison with the original blurry image, the information (e.g., the Chinese characters in the right box) is still difficult to identify. The deblurred result of [35] is shown in Figure 4d. Although the method [35] performs well in identifying some content information, various noise and line artifacts can be found in the output image, resulting in an unnatural visual effect. Motion blur is little removed by Dong’s method [26], as shown in Figure 4e, and detail information is unrevealed. In Figure 4f, although some contents can be recognized, however, the blur effects are less removed (i.e., the Chinese characters in right side of the gate). The proposed method outperforms other algorithms in removing blur and producing results with more complete detail information and fewer artifacts.

From Figure 5, we can see that Xu’s method [33] can achieve the most satisfying result among all competing algorithms since the highest degree of identification and the elimination of some small-scale blur (e.g., geometric form in the red box in Figure 5b). However, ringing artifact is still the biggest problem existing in the deblurring result. The results from Figure 5c–e show that very few blur effects are removed and the images are still blurry. In Figure 5c,e, both method [34] and method [26] fail to recover fine details in the restored images. The deblurring result of method [35] is not visually desirable since artefacts can be observed. In Figure 5f, since the Chinese characters are relative larger, the structures can be reconstructed for recognition. However, there are still large amounts of ringing artefacts. The proposed method is highly effective in removing blur and representing fine detail information to produce favorable results.

Experimental comparisons using a synthetic image are shown in Figure 6. As shown in Figure 6c,d, the Wiener filter and Xu’s method [33] can better remove blur at the strong edges because minimization of the mean square error and step-edge properties can benefit the process of deblurring. However, they fail to suppress ringing artifacts, especially in areas near step edges. The deblurring result of Shen’s metnod [34] shows that it is difficult for local-contrast based algorithms to handle synthetic image deblurring. Yang’s method [35] and Dong’s method [26] perform better than other compared algorithms in removing blur and suppressing ringing artefacts. However, some other visual artefacts (e.g., noise) can be observed in Figure 6f,g. In Figure 6h, ringing artefacts can be detected in edge areas. It is clear that the proposed method exhibits the highest synthetic image deblurring performance among all compared algorithms.

PSNR and SSIM are two powerful perceptual quality metrics that have been widely applied to quantitatively evaluate image restoration algorithm performance. Table 1 shows the PSNR and SSIM values of each algorithm—the highest belonging to the proposed method.

## 4. Conclusions

In this paper, we present an ensemble dictionary learning model with low-rank constraint for single image deblurring. The ensemble dictionary set is offline-learned and online-utilized as prototypes for each input blurred image patch to be represented. Based on our observation and analysis for two related issues, the proposed low-rank embedded regularization is very effective for linking structural features with our learned ensemble dictionary set and hence can improve the performance of sparse representation-based image deblurring application. Two issues assure the consistent robustness between textured background and foreground. First, since the image patches that we use for training ensemble dictionary contain various of structures and contents including not only foreground but also background. It implies that the structures of the background can also be well represented. Second, one of the advantages of sparse representation-based method is that the deblurring performance makes no difference between foreground and background as long as the same dictionary is given. Because the patches both in background and foreground are simultaneously extracted and represented in the same manner. The optimization can be solved by decomposing the model into two sub-problems. Each sub-problem has a closed-form solution. The experimental results show that the proposed method outperforms other image restoration algorithms, both qualitatively and quantitatively. However, for those blurred images whose blur strength is very strong, since the size of image patch is relative small (i.e., 8×8), extracting structural features itself is hard to achieve, besides, the connection between structural features and sharp primitives is week. For these reasons, the performance for severe blurred images is less effective. Further research is needed to handle those problems. 

## Figures and Tables

**Table 1:** Quantitative comparison by peak signal-to-noise ratio (PSNR) and structural similarity index (SSIM) evaluation metrics.

Algorithm	motion0015			motion0105			out_of_focus0122			out_of_focus0290	
	PSNR	SSIM		PSNR	SSIM		PSNR	SSIM		PSNR	SSIM
Xu’s method	26.03	0.89		23.18	0.81		25.54	0.89		15.05	0.58
Shen’s method	29.65	0.94		36.46	0.97		30.26	0.94		33.34	**0.96**
Yang’s method	27.82	0.84		28.88	0.82		29.51	0.87		29.51	0.87
Dong’s method	29.65	0.88		30.86	0.87		29.98	0.93		27.91	0.87
CSR method	30.02	0.91		31.27	0.89		32.13	0.95		30.27	0.93
Wiener filter	8.99	0.04		12.53	0.13		9.81	0.09		10.71	0.11
Proposed method	**31.57**	**0.95**		**40.21**	**0.97**		**32.19**	**0.96**		**35.40**	0.95
